# Biodiversity and Hemolytic Toxicity of the Genus *Heterocapsa* (Dinophyceae) in the Beibu Gulf, China

**DOI:** 10.3390/md22110514

**Published:** 2024-11-14

**Authors:** Yixiao Xu, Nina Dzhembekova, Kirsty F. Smith, Haifeng Gu, Uwe John, Huanda Xie, Yujuan Wen, Miao Wu

**Affiliations:** 1 Key Laboratory of Environment Change and Resources Use in Beibu Gulf, Ministry of Education, Nanning Normal University, Nanning 530001, China; xiehuanda@163.com (H.X.); wenyujuan1021@163.com (Y.W.); wumiao_2020@163.com (M.W.); 2 Guangxi Beibu Gulf Intelligent Marine Ranching Engineering Research Center, Nanning Normal University, Nanning 530001, China; 3 Institute of Oceanology-Bulgarian Academy of Sciences, 9000 Varna, Bulgaria; sonata_bg@yahoo.com; 4 Cawthron Institute, Nelson 7010, New Zealand; kirsty.smith@cawthron.org.nz; 5 Third Institute of Oceanography, Ministry of Natural Resources, Xiamen 361005, China; guhaifeng@tio.org.cn; 6 Alfred-Wegener-Institute Helmholtz Centre for Polar and Marine Research, 27570 Bremerhaven, Germany

**Keywords:** phylogeny, taxonomy, toxicity, erythrocyte lysis assay, dinoflagellate, harmful algal blooms, Beibu Gulf

## Abstract

The dinoflagellate genus *Heterocapsa* includes several widely distributed and potentially toxic species associated with Harmful Algal Blooms (HABs), particularly affecting the Western Pacific Ocean. To reveal the biodiversity of *Heterocapsa* in Beibu Gulf, six strains were morphologically characterized using light and scanning electron microscopy, while large subunit rDNA (LSU rDNA) and internal transcribed spacer (ITS) were sequenced for phylogenetic analysis through maximum likelihood and Bayesian inferences. Two strains (BGERL169, BGERL170) were identified as *Heterocapsa philippinensis* ribotype I, three (BGERL171-BGERL173) as a new *Heterocapsa philippinensis* ribotype II, and one strain (BGERL174) as *Heterocapsa pseudotriquetra*. Cells of *H. philippinensis* were ovoid to spherical, yellowish-brown, with reticulate chloroplasts, and had a sausage-shaped nucleus positioned longitudinally along the dorsal side of the cell, and the theca was arranged in Po, cp, X, 5′, 3a, 7″, 6c, 5s, 5‴, 2⁗. Additionally, BGERL169 and BGERL171 showed no hemolytic toxicity in rabbit erythrocyte lysis assays. To the best of our knowledge, this research provides the first morphological and phylogenetic analysis of *H. philippinensis*, including the identification of a new ribotype, as well as the discovery of *H. pseudotriquetra* in Chinese waters. The findings contribute to the understanding of *Heterocapsa* species biogeography and toxicity in Chinese waters, offering valuable data for future HAB monitoring in Beibu Gulf.

## 1. Introduction

The genus *Heterocapsa* belongs to the phylum Dinophyta, class Dinophyceae, order Peridiniales, and family Heterocapsaceae. This dinoflagellate, known for causing harmful algal blooms (HABs), is widely distributed in temperate, subtropical, and tropical marine environments [1,2]. The taxonomic history of the genus is notably complex. In 1883, Stein renamed *Glenodium triquetrum* Ehrenberg to *Heterocapsa triquetra* Ehrenberg, establishing the genus *Heterocapsa*; and later, Loeblich designated *H. triquetra* as the type species of the genus [3,4]. However, over 100 years later, phycologists discovered that the originally defined type species, *H. triquetra*, actually belonged to the genus *Kryptoperidinium* rather than *Heterocapsa* [5,6]. Additionally, the other two species Stein initially described under *Heterocapsa*, *Heterocapsa quadridentata* F. Stein and *Heterocapsa umbilicata* F. Stein, were not *Heterocapsa*, with the former species now belonging to *Blixaea* Gottschling and the latter species to an undefined genus [7]. To preserve the genus *Heterocapsa* and avoid extensive nomenclatural changes, it was proposed reassigning *Heterocapsa steinii*, discovered in the Baltic Sea where the type species of *Heterocapsa* was originally established, as the new type species for the genus [7,8]. This proposal was accepted by the Nomenclature Committee for Algae in 2020 [9]. To date, 26 species of *Heterocapsa* have been recorded.

The cell of *Heterocapsa* is spindle-shaped, centrally symmetrical, and covered with minute three-dimensional body scales, with yellow-brown chloroplasts and thecal plate formula of Po, cp, X, 4–5′, 2–3a, 6–7″, 6c, 5s, 5‴, 2⁗ [2,8]. Morphological identification primarily depends on the shape of the cell, the relative position and structure of the nucleus and pyrenoid, as well as the ultrastructure of the body scales [2]. Among these characteristics, body scale features are regarded as reliable indicators for distinguishing species within the genus *Heterocapsa* [1,10]. Moreover, rDNA markers including the large subunit rDNA (LSU rDNA) and internal transcribed spacer (ITS) region also play a key role in *Heterocapsa* spp. systematics and has been widely used for species identification [11].

In addition to forming HABs by mass occurrence, certain species of *Heterocapsa* also produce hemolytic toxins that cause mass mortality in marine organisms. The first *Heterocapsa* species reported to produce hemolytic toxins is *Heterocapsa circularisquama*, which has been linked to significant bivalve mortality events in Japan and the Western Pacific region [12,13]. *Heterocapsa bohaiensis*, discovered in the coast of Bohai Sea of China, has also been found to exhibit hemolytic toxicity, causing mass mortality of cultured prawns and larvae of Chinese mitten handed crabs in aquaculture ponds [14]. In 2019, during a HAB outbreak dominated by *Skeletonema*, *Prorocentrum*, *Gymnodinium*, and *Heterocapsa* species in Sansha Bay, Fujian, China, *Heterocapsa horiguchii*, *Heterocapsa* cf. *pygmaea*, and *Heterocapsa* cf. *niei* were isolated from the seawater [15]. These three species were confirmed to be lethal to brine shrimp *Artemia salina* [15]. In addition to hemolytic toxicity, the ichthyotoxicity of *Heterocapsa* and its cytotoxic effects on other marine organisms like microzooplankton rotifer and *Artemia* nauplii, towards cells from the gills of rainbow trout, and even bactericidal activity have also been reported [11,16,17,18].

The study area is the Beibu Gulf, located in the northwest of the South China Sea, is characterized by vast shallow coastal waters, making it an ideal area for marine aquaculture in China. However, in the Beibu Gulf the frequency, duration, and extent of HABs have significantly increased over the past two decades, exhibiting not only damages to the marine ecosystem but also negative impact on coastal aquaculture and human health [19]. Although no *Heterocapsa* blooms have been recorded in the Beibu Gulf to date [20], this species is recognized as one of the main toxic and HAB-causing organism in the Chinese and Western Pacific waters. Nearby waters in Hong Kong have already reported blooms of *H. circularisquama* [21,22]. Therefore, it is crucial to identify the species composition and toxicity of *Heterocapsa* in the Beibu Gulf. In this study, six *Heterocapsa* strains were isolated, and species were identified using a combination of light microscopy, scanning electron microscopy (SEM), and phylogenetic analysis. Hemolytic toxicity was also determined on two *Heterocapsa* strains.

## 2. Results

### 2.1. Morphological Characteristics

**Figure 1:**
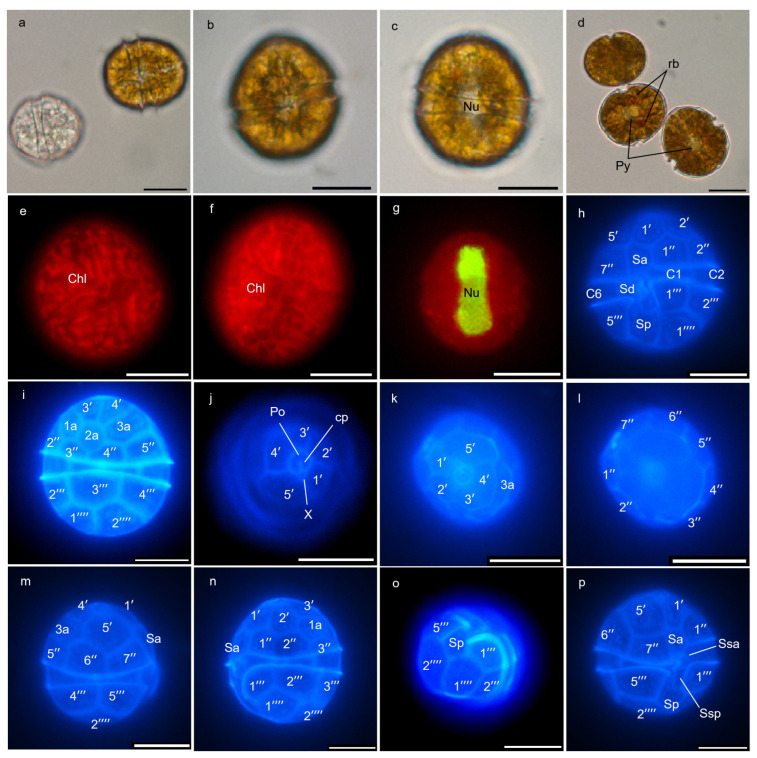
Light microscope images of *Heterocapsa* strain BGERL169. (**a**): live cells and empty theca, (**b**): ventral view, (**c**): dorsal view, (**d**): red body (rb) and pyrenoid (Py), (**e**–**p**): fluorescence microscope images, with chloroplast (Chl), (**g**): nucleus (Nu), (**h**): ventral view, (**i**): dorsal view, (**j**–**l**): apical view, (**m**): right lateral view, (**n**): left lateral view, (**o**): antapical view, (**p**): ventral view. Po: apical pore plate, cp: cover plate, X: canal plate, 1′–5′: apical plates, 1a–3a: anterior intercalary plates, 1″–7″: precingular plates, C1, C2, and C6: cingular plates, Sa, Ssa, Sd, Sp, and Ssp: sulcal plates, 1‴–5‴: postcingular plates, 1⁗–2⁗: antapical plates. Scale bar: 10 μm.

**Figure 2:**
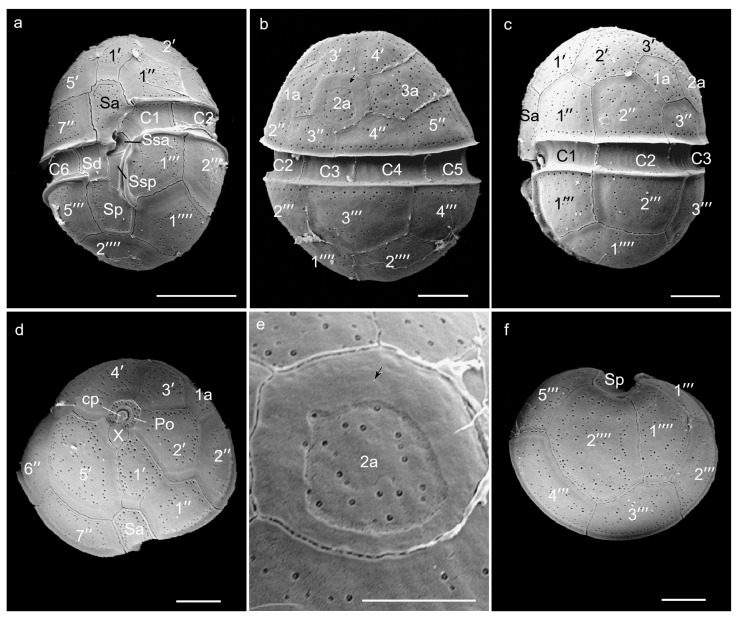
Scanning electron microscope images of *Heterocapsa* strain BGERL169. (**a**): ventral view, (**b**): dorsal view, (**c**): left lateral view, (**d**): apical view, (**e**): the second anterior intercalary plate, (**f**): antapical view. Po: apical pore plate, cp: cover plate, X: canal plate, 1′–5′: apical plates, 1a–3a: anterior intercalary plates, 1″–7″: precingular plates, C1–C6: cingular plates, Sa, Ssa, Sd, Sp, and Ssp: sulcal plates, 1‴–5‴: postcingular plates, 1⁗–2⁗: antapical plates. Scale bar: 5 μm (**a**–**d**,**f**) and 3 μm (**e**).

**Figure 3:**
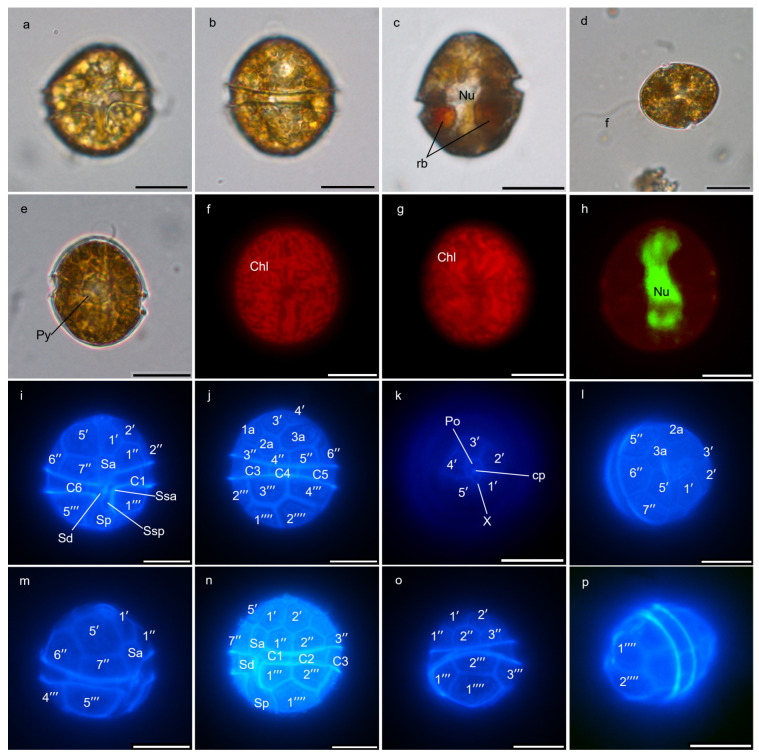
Light microscope images of *Heterocapsa* strain BGERL171. (**a**): ventral view, (**b**): dorsal view, (**c**): nucleus (Nu) and red body (rb), (**d**): flagellum (f), (**e**): pyrenoid (Py), (**f**–**p**): fluorescence microscope images, (**f**,**g**): chloroplast (Chl), (**h**): nucleus (Nu), (**i**): ventral view, (**j**): dorsal view, (**k**,**l**): apical view, (**m**): apical-right lateral view, (**n**): ventral-left lateral view, (**o**): dorsal view, (**p**): partial antapical view. Po: apical pore plate, cp: cover plate, X: canal plate, 1′–5′: apical plates, 1a–3a: anterior intercalary plates, 1″–7″: precingular plates, C1–C6: cingular plates, Sa, Ssa, Sd, Sp, and Ssp: sulcal plates, 1‴–5‴: postcingular plates, 1⁗–2⁗: antapical plates. Scale bar: 10 μm.

**Figure 4:**
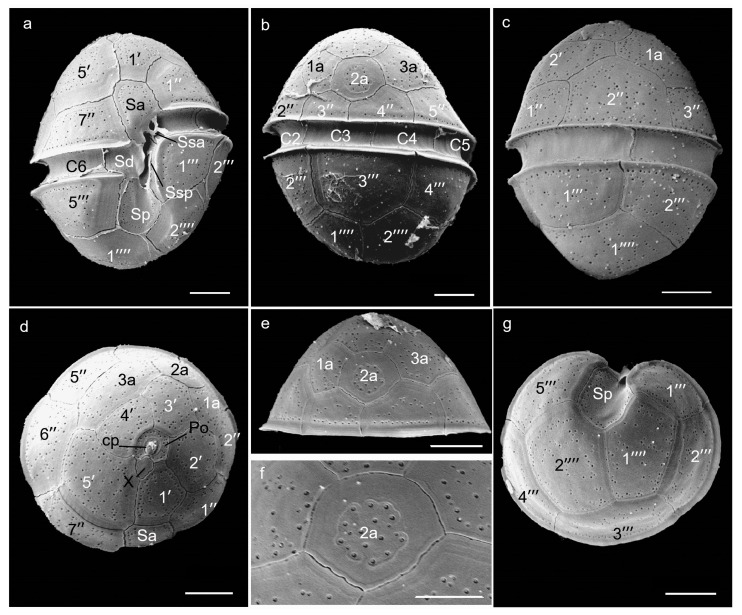
Scanning electron microscope images of *Heterocapsa* strain BGERL171. (**a**): ventral view, (**b**): dorsal view, (**c**): left lateral view, (**d**): apical view, (**e**,**f**): the second anterior intercalary plate (2a), (**g**): antapical view. Po: apical pore plate, cp: cover plate, X: canal plate, 1′–5′: apical plates, 1a–3a: anterior intercalary plates, 1″–7″: precingular plates, C1–C6: cingular plates, Sa, Ssa, Sd, Sp, and Ssp: sulcal plates, 1‴–5‴: postcingular plates, 1⁗–2⁗: antapical plates. Scale bar: 5 μm (**a**–**e**,**g**) and 3 μm (**f**).

Both strains, BGERL169 and BGERL171, were unicellular and brownish-yellow in color, with ovoid and spherical cells (Figure 1, Figure 2, Figure 3 and Figure 4). The length of BGERL169 was 20.0 ± 3.9 μm, and the width was 16.7 ± 2.6 μm (*n* = 30), while BGERL171 had a length of 21.0 ± 3.2 μm and a width of 17.6 ± 3.7 μm (*n* = 30) (Figure 1, Figure 2, Figure 3 and Figure 4). Both BGERL169 and BGERL171 exhibited similarly sized hemispherical epitheca and hypotheca (Figure 1a–d and Figure 3a–e), with a relatively wide and deep cingulum that exhibited a distinct left-hand displacement (descending). The distance between the two ends of the cingulum was approximately equal to its width (Figure 1a,b and Figure 3a). The nucleus was sausage-shaped and located at the periphery of the dorsal side of the cell, while the pyrenoid was centrally located, with two conspicuous red bodies (Figure 1d,g and Figure 3c,e).

The plate arrangement was Po, cp, X, 5′, 3a, 7″, 6c, 5s, 5‴, 2⁗ (Figure 1, Figure 2, Figure 3 and Figure 4). The apical pore complex (APC) featured a nearly pentagonal horseshoe-shaped apical pore plate (Po), with a prominent globular cover plate (cp) in the center of the Po plate, connected to the canal plate (X) (Figure 2d and Figure 4d). A circular ridge with a depression corresponding to the apical pore plate was observed (Figure 2d and Figure 4d). The irregular-shaped canal plate (X) was located between the first and fifth apical plates (1′ and 5′) (Figure 2d and Figure 4d). Among the five apical plates (1′–5′), the fifth apical plate was the largest (Figure 2d and Figure 4d). Of the three anterior intercalary plates (1a–3a), the second anterior intercalary plate (2a) had a wide and thick margin, lacking pore structures (Figure 2b,e and Figure 4e,f). Seven precingular plates (1″–7″) connected to the cingulum, which was composed of six cingulum plates (C1–C6); C1 and C6 were located on the ventral side, while the remaining cingulum plates were positioned on the lateral and dorsal sides (Figure 2a,b and Figure 4a–c). 

Among the five sulcal plates (Sa, Ssa, Sd, Sp, Ssp), the Sa plate extended to the epitheca and connected with the apical plate 1′ and 5′, and precingular plates 1″ and 7″, while the Sd plate connected to the C6 plate on the right side. Two smaller sulcal plates (Ssa, Ssp) were located along the edge of the sulcal plates, and the Sp plate extended to hypotheca, connecting with the postcingular plates 1‴ and 5‴, as well as antapical plates 1⁗ and 2⁗ (Figure 2a and Figure 4a). The hypotheca was made up of five postcingular plates (1‴–5‴) and two antapical plates (1⁗–2⁗) (Figure 1, Figure 2, Figure 3 and Figure 4).

Therefore, the morphological characteristics of the two strains, BGERL169 and BGERL171, align with those of *H. philippinensis*, particularly the longitudinally elongated sausage-shaped nucleus and the circular second anterior intercalary plate (2a) [2]. Thus, we suggest that these two strains belong to *H. philippinensis* or its closely related morphotypes.

### 2.2. Phylogenetic Characteristics

**Figure 5:**
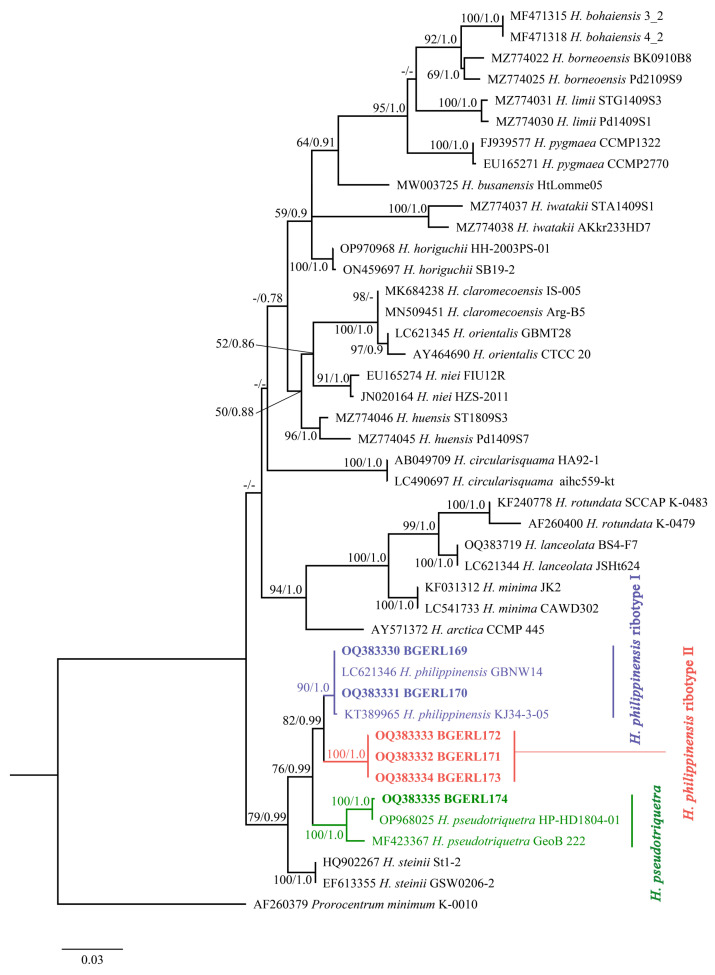
Phylogenetic tree based on LSU rDNA (bootstrap and posterior probability values below 50 and 0.75 are indicated by “-” in the tree).

**Figure 6:**
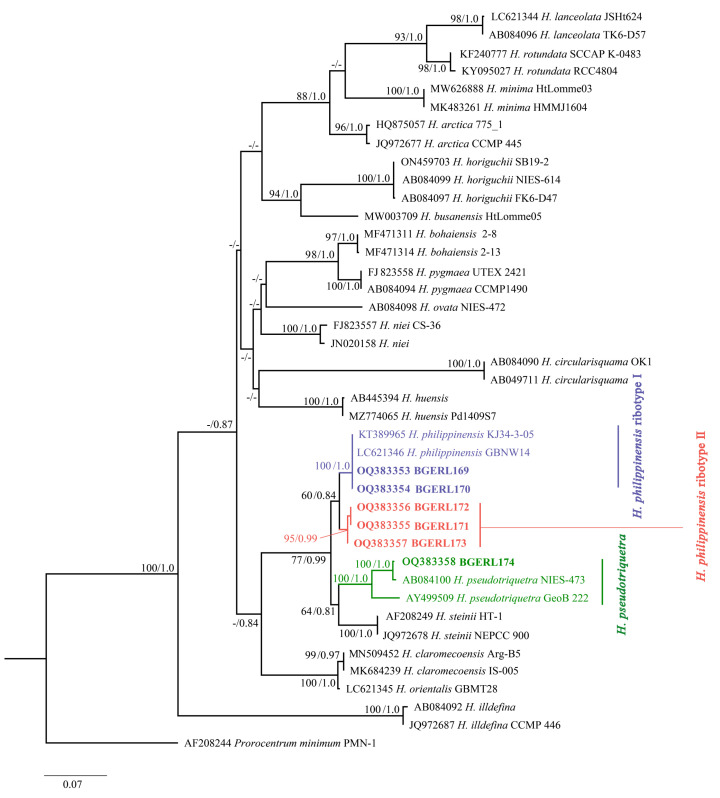
Phylogenetic tree based on ITS (bootstrap and posterior probability values below 50 and 0.75 are indicated by “-” in the tree).

The average lengths of LSU rDNA and ITS sequences for six *Heterocapsa* strains from the Beibu Gulf were 1400 bp and 638 bp, respectively. After trimming and alignment, the sequence lengths used for phylogenetic analysis including gaps were 949 bp and 654 bp, respectively. In this study, *Prorocentrum minimum* was used as the outgroup to construct ML and BI phylogenetic trees like the previous studies [2,15]. The tree topologies generated by both methods were largely consistent; thus, the ML tree was presented here (Figure 5 and Figure 6).

The LSU rDNA phylogenetic tree revealed that strains BGERL169 and BGERL170 from the Beibu Gulf clustered together with *H. philippinensis* (LC621346 and KT389965), with support values of 90/1.0. Strains BGERL171-BGERL173 formed a separate clade with a support value of 100/1.00. This clade did not cluster directly with any other identified *Heterocapsa* species but formed a sister clade to the *H. philippinensis* branch, which included BGERL169 and BGERL170, with support values of 82/0.99. Additionally, BGERL174 from the Beibu Gulf initially clustered with the strain *H. pseudotriquetra* HP-HD1804-01, with support values of 100/1.00, and this branch subsequently formed a sister clade with *H. pseudotriquetra* strain GeoB 222 in support values of 100/1.00 (Figure 5).

The ITS phylogenetic tree similarly showed that strains BGERL169 and BGERL170 from the Beibu Gulf clustered with *H. philippinensis* (LC621346 and KT389965), with support values of 100/1.00. Strains BGERL171-BGERL173 clustered together with a support value of 95/0.99, and, like the LSU rDNA phylogenetic tree, this clade did not cluster directly with any other identified *Heterocapsa* species but formed a sister clade to the *H. philippinensis* branch, with support values of 60/0.84. Strain BGERL174 clustered with two *H. pseudotriquetra* strains (AB084100 and AY499509), with support values of 100/1.00 (Figure 6).

In the LSU rDNA phylogenetic analyses, the interspecies genetic distances among *Heterocapsa* species ranged from 0.016 (between *H. claromecoensis* and *H. orientalis*) to 0.778 (between *H. bohaiensis* and *H. circularisquama*). The genetic distance between strains BGERL171-BGERL173 and the closest *H. philippinensis* branch was 0.043, which was greater than the distance between *H. claromecoensis* and *H. orientalis* (0.016) and *H. lanceolata* and *H. rotundata* (0.036). In the ITS phylogenetic tree, the interspecies genetic distances ranged from 0.005 (between *H. orientalis* and *H. claromecoensis*) to 0.765 (between *H. circularisquama* and *H. illdefina*). The genetic distance between strains BGERL171-BGERL173 and the closest *H. philippinensis* branch was 0.022, which was greater than the interspecies distance between *H. orientalis* and *H. claromecoensis* (0.005). Similarly, the intraspecies genetic distances in the LSU rDNA and in the ITS phylogenetic tree for *Heterocapsa* spp. varied from 0–0.039 and 0–0.040, respectively. Therefore, based on genetic distance alone, it was clear that the strains BGERL171-BGERL173 formed a distinct clade and ribotype from *H. philippinensis*. However, no obvious morphological differences from the typical characteristics of *H. philippinensis* were identified.

### 2.3. Hemolytic Toxicity

Only strains BGERL169 (*H. philippinensis* ribotype I) and BGERL171 (*H. philippinensis* ribotype II) were subjected to hemolytic toxicity assays. The result showed that no hemolytic toxicity was detected in either the methanol or chloroform fractions of these two strains.

## 3. Discussion

Due to the relatively small size and morphological similarities of most *Heterocapsa* species, as well as intraspecies variability in cell shape even during culture maintenance [10], identifying *Heterocapsa* species using light microscopy remains difficult. In this study, the plate pattern of *Heterocapsa* strains BGERL169 and BGERL171, Po, cp, X, 5′, 3a, 7″, 6c, 5s, 5‴, 2⁗, was the most common plate arrangement observed among *Heterocapsa* species [1,23]. Their morphological characteristics matched those of *H. philippinensis* defined in the literature [2], particularly the rectangular, sausage-shaped nucleus located in the middle of the dorsal side, and the thick-edged, poreless circular second anterior intercalary plate (2a). Benico et al. [2] emphasized that the distinctive features of the second anterior intercalary plate (2a) in *H. philippinensis* were unique among known *Heterocapsa* species, making it a key trait when defining this species in morphology. Therefore, strains of BGERL169 and BGERL171 from the Beibu Gulf should be very closely related with *H. philippinensis*. The LSU rDNA and ITS phylogenetic analysis further confirmed BGERL169–170 belonged to the species *H. philippinensis*. 

However, the ribosomal sequences of BGERL171–173 differed somewhat from those of *H. philippinensis*, forming an independent clade in the phylogenetic tree, with strong bootstrap support and Bayesian inference posterior probabilities values of 100/1.0 and 95/0.99, respectively. The genetic distances also suggest that these three strains may represent interspecies divergence from *H. philippinensis*. Therefore, strains BGERL171-BGERL173 are tentatively defined as *H. philippinensis* ribotype II, while the type species *H. philippinensis* is designated as *H. philippinensis* ribotype I. Cryptic diversity, with a clearly well-supported new ribotype, is revealed through LSU rDNA and ITS phylogenetic analysis. However, due to the lack of morphological differences, no taxonomic arguments can be provided for establishing a new species. To clarify their taxonomy, further research, including more strains of these ribotypes and their ultrastructural features, such as body scale characteristics, is necessary.

This study is the first to report *H. philippinensis* and *H. pseudotriquetra* in Chinese waters. *H. philippinensis* is a newly described species, first discovered in the Philippines [2]. The *H. philippinensis* strains BGERL169–170 from the Beibu Gulf were closely related to the type strain GBNW14 and the strain KJ34-3-05 from the South China Sea [2,24]. Strain KJ34-3-05 was recorded in 2015, and its ribosomal sequence was 1583 bp comprising part of the 18S rDNA, the complete ITS region, and part of the 28S rDNA. However, this strain lacked a morphological description and was initially identified as *H. triquetra*. Later, Benico et al. [2], when defining *H. philippinensis*, found that strain KJ34-3-05, despite the absence of morphological data, had LSU and ITS sequences that were 100% identical to the type strain GBNW14 of *H. philippinensis*. This suggests that *H. philippinensis* had already been present in Chinese waters, although it had not been properly reported. Our study confirms the presence of *H. philippinensis* in Chinese waters, providing crucial molecular and morphological data that rectifies previous misidentifications and enhances our understanding of the species’ distribution range. Additionally, *H. pseudotriquetra* was first recorded by Iwataki [10], but this species has been infrequently recognized. Currently, only three strains of *H. pseudotriquetra* are available in the NCBI GenBank database (HP-HD1804-01, GeoB 222, and NIES473). Strain BGERL174 from the Beibu Gulf showed a high similarity to these strains, exhibiting 100%/1.0 similarity to strains HP-HD1804-01 and GeoB 222 in LSU rDNA, and 100%/1.0 similarity to strains NIES473 and GeoB 222 in ITS. Although this study lacked morphological data of BGERL174, the LSU and ITS phylogenetic results strongly suggest that it belongs to *H. pseudotriquetra*.

Hemolysin is one of harmful toxins secreted by HAB species [25,26]. Among *Heterocapsa* species, *H. circularisquama*, one of the earliest discovered, has caused particularly serious problems in the Western Pacific Ocean, and its hemolytic toxicity has been extensively studied. Previous research showed hemolytic toxicity of *H. circularisquama* was associated with its cell density, the duration of experimental cultivation, and the type of *Heterocapsa* cells used in experiments (e.g., live cell suspensions, lysed cell extracts, or de-thecated cell solutions) [13,16,27,28,29]. *H. circularisquama* contains the hemolytic toxin (H2-a) that has similar chemical structure to pyropheophorbide *a* methyl ester, a well-known photoactive hemolytic agent [28]. Even at low density (5 cells mL^−1^) and low temperature (15 °C), exposure to *H. circularisquama* can induce broad cytotoxic effects in the vital organs of Mediterranean mussels (*Mytilus galloprovincialis*) [30]. Bloom of *H. circularisquama* has caused serious economic losses in mariculture in Japan [31,32,33]. Although this species has not been recorded in the Beibu Gulf, nearby waters in Hong Kong have reported recurrent blooms since 1986 [21,22]. Investigating the distribution and potential toxicity of *H. circularisquama* in the Beibu Gulf is essential.

Hemolysins from *Heterocapsa* spp. may be photosensitizing porphyrin derivatives, aligns with the observed effect of light on the hemolytic toxicity, i.e., hemolytic activity of *Heterocapsa* is present under light conditions but disappears in the dark [29,34]. In addition to *H. circularisquama*, a similar light-dependent relationship with hemolytic activity has also been recently described in *Heterocapsa horiguchii*, *Heterocapsa* cf. *pygmaea*, and *Heterocapsa* cf. *niei*, isolated from a HAB event in Sansha Bay, Fujian, China [15]. However, another toxic species of *H. bohaiensis* isolated in 2018 from Bohai Sea, China, had hemolytic toxicity regardless of light or dark conditions, and the toxicity was primarily correlated with cell density [17,35]. Prior to this study, there has been no research on the hemolytic toxicity of *H. philippinensis*. In this study, hemolytic activity of *H. philippinensis* ribotype I (BGERL169) and *H. philippinensis* ribotype II (BGERL171) was determined under light, with cell densities of (1.1–1.9) × 10^4^ cells mL^−1^, which is comparable to the *Heterocapsa* densities used in the previous publications. Despite this, no hemolytic toxicity was detected. Therefore, it proves that the studied *H. philippinensis* strains tested here, like some other species such as *H. triquetra* [13,36], do not produce hemolytic toxins. It remains to be tested with other strains from different localities if all representatives of this species are non-toxic/lytic or under which conditions.

## 4. Materials and Methods

### 4.1. Culture Resources

Phytoplankton samples were collected from Lianzhou Bay and Tieshan Harbor in the Beibu Gulf of China, on January, 2022. Sample collection, storage, and transportation were carried out followed the specification for marine monitoring of China (GB17378.1-2007) [37]. In the laboratory, *Heterocapsa* cells were isolated using capillary pipettes under the inverted microscope (TS100, Nikon Corporation, Tokyo, Japan). Six *Heterocapsa* strains were successfully established and maintained in modified K medium (without Si), 23 °C, salinity of 32‰, light intensity of 150 μmol m^−2^ s^−1^, and a light–dark cycle of 12 h:12 h (Table 1). Phylogenetic analysis of rDNA was conducted on all six *Heterocapsa* strains. However, due to culture loss during maintenance, only strains BGERL169 and BGERL171 were subjected to microscopic observation and hemolytic toxicity assays.

### 4.2. DNA Extraction and Amplification

*Heterocapsa* cultures in the exponential growth phase were collected by centrifugation, and DNA was extracted using BioFastSpin DNA extraction kit (Bioer Technology, Hangzhou, China) following the protocol of the manufactor. For the amplification of the LSU rDNA sequence, the forward primer was 28S-D1R (ACCCGCTGAATTTAAGCATA), and the reverse primer was 28S-1483R (GCTACTACCACCAAGATCTGC) [38,39]. For the ITS sequence amplification, the forward primer was ITSA (CCTCGTAACAAGGHTCCGTAGGT), and the reverse primer was ITSB (CAGATGCTTAARTTCAGCRGG) [40,41]. The total PCR reaction volume was 40 μL, containing 20 μL of 2× Es Taq Master Mix, 1 μL each of the forward and reverse primers, 1 μL of DNA template, and 17 μL of ddH_2_O. PCR was performed using a Biometra Easy Cycler Gradient thermal cycler (Analytik Jena GmbH+Co., Jena, Germany) with an initial denaturation step at 94 °C for 3 min, followed by 35 amplification cycles. Each cycle consisted of a denaturation step at 94 °C for 30 s, an annealing step at 53 °C for 30 s for LSU rDNA and 56 °C for 30 s for ITS, and an extension step at 72 °C for 30 s. The reaction concluded with a final elongation at 72 °C for 5 min. The PCR products were sequenced using the Sanger method in Beijing Tsingke Biotech Co., Ltd., Beijing, China.

### 4.3. Morphology Observation

For light microscopy observation, *Heterocapsa* cells in the exponential growth phase were observed under a fluorescence microscope (Ni-U, Nikon Corporation, Tokyo, Japan) using an oil immersion objective at 100× magnification. The cell morphology and cell size were analyzed using the NIS-Elements D (v4.50.00 ). Chloroplasts were observed under blue light excitation at 510–560 nm. The nuclei of *Heterocapsa* cells were stained with SYBR Green I (Shanghai yuanye Bio-Technology Co., Ltd., Shanghai, China) and observed under blue light excitation at 450–490 nm. The thecal plates were stained with Fluorescent Brightener (Shanghai yuanye Bio-Technology Co., Ltd., Shanghai, China) and also observed under blue light excitation at 330–380 nm.

For SEM observation, *Heterocapsa* cells in the exponential growth phase were fixed for 4 h by adding 50% glutaraldehyde solution (Sigma-Aldrich Ltd., St. Louis, MO, USA), then filtered through a 3 μm polycarbonate membrane (Merck Millipore Ltd., Darmstadt, Germany) for further desalination and dehydration treatment. For desalination, sterile seawater was used, and the cells were gradually exposed to a salinity gradient of 90%, 70%, 50%, 30%, 10%, 0%, and 0%, with each step lasting 15 min. The cells were subsequently dehydrated using ethanol in a concentration gradient of 10%, 30%, 50%, 70%, 90%, 100%, and 100%, with each step also lasting 15 min. The samples were subsequently dried using a critical point dryer (Quorum K850, Quorum Technologies Ltd., Laughton, UK), sputter-coated with gold in a Cressington 108auto coater (Cressington Scientific Instruments Ltd., Watford, UK), and observed and made pictures in SEM (TM-1000, Hitachi High-Technologies Corporation, Tokyo, Japan). 

### 4.4. Phylogenetic Analysis

The LSU rDNA and ITS sequences of the six *Heterocapsa* strains were used for phylogenetic analyses (Table 1). Therefore, similar sequences were searched using BLAST and downloaded from NCBI database. Sequences were aligned using MUSCLE algorithm and genetic distances were calculated in MEGA-X (v10.1.8). The evolutionary models were determined using jModelTest 2 (v0.1.11) [42,43]. Phylogenetic trees were constructed using the maximum likelihood method in RAxML (v8.2.X) [44], with 1000 bootstrap replicates to assess branch confidence. The GTR + G + I and GTR + G evolutionary models were applied for the LSU rDNA and ITS sequence, respectively. Additionally, Bayesian inference trees were constructed using MrBayes 3.2.7a [45]. The TIM3 + I + G and GTR + G evolutionary models were used for the LSU rDNA and ITS sequence, respectively. Bayesian analysis was run for 2 million generations with four Markov chains, sampling every 200 generations, and convergence diagnostics were calculated every 1000 generations. The temperature constant was set to 0.2, and a burnin percentage of 25% was applied. Finally, the phylogenetic trees were viewed and edited using FigTree (v1.4.4) and Adobe Illustrator (2020 v24.1.1), and displayed based on the maximum likelihood tree.

### 4.5. Hemolytic Toxicity Assay

The assay followed the protocol by Eschbach et al. [46]. Cells of BGERL169 (1.9 × 10^4^ cells mL^−1^, totaling 1.8 × 10^7^ cells) and BGERL171 (1.1 × 10^4^ cells mL^−1^, totaling 9.9 × 10^6^ cells) in the exponential growth phase were centrifugally harvested and resuspended in a chloroform: methanol: water mixture (13:7:5) to perform ultrasonic disruption in an ice bath. The supernatant was centrifugally collected and stored overnight at 4 °C to allow for methanol and chloroform layers separation. These two layers were then transferred into separate 15 mL centrifuge tubes. After drying the methanol and chloroform fractions using a nitrogen evaporator, the residues were dissolved in 1 × PBS buffer solution, and further filtered through a 0.22 μm membrane to obtain the crude hemolysin. For hemolytic toxicity measurement, 0.5 mL of the crude hemolysin extract and 0.5 mL of 0.5% rabbit erythrocyte solution were added to a 2 mL tube (A_s_). The following controls were used: 0.5 mL of 1 × PBS buffer and 0.5 mL of 0.5% rabbit erythrocyte solution as a negative control (A_a_); 0.5 mL of the crude hemolysin extract and 0.5 mL of PBS buffer as a blank control (A_b_); and 0.5 mL of 1% non-ionic surfactant Triton X-100 (Sigma-Aldrich Ltd., St. Louis, MO, USA) and 0.5 mL of 0.5% rabbit erythrocyte solution as a positive control (A_c_). Each reaction was carried out in triplicate. The mixtures were incubated at 25 °C with a light intensity of 100 μmol m^−2^ s^−1^ for 6 h. The absorbance value was measured at 405 nm in a microplate reader and used to calculate the percentage of hemolytic activity (P) according to the following formula [47].

(1) $$P=\left(A_{s}-A_{a}-A_{b}\right)\times 100/A_{c}$$

## 5. Conclusions

In this study, six strains of *Heterocapsa* were isolated from the Beibu Gulf, China. Based on a combination of morphological characteristics and molecular phylogenetic analyses, these strains were assigned to *H. philippinensis* ribotype I (the type species of *H. philippinensis*), *H. philippinensis* ribotype II, and *H. pseudotriquetra*. This study performed the first morphological and phylogenetic description of *H. philippinensis* in Chinese waters, as well as the first assessment of its hemolytic toxicity. *H. pseudotriquetra* is also newly recorded in Chinese waters, while *H. philippinensis* ribotype II is the first discovery of this genotype globally, potentially representing a new species.

This study provides essential biological data for HAB prevention and control in the Beibu Gulf and enhances our understanding of the distribution and toxicity of *Heterocapsa* species in Chinese waters. While culturing is a reliable approach for studying species biodiversity, it presents challenges in isolating dinoflagellate cells like *Heterocapsa* from natural seawater. Integrating high-throughput sequencing, such as metabarcoding, with culturing methods will enable a comprehensive understanding of *Heterocapsa* species composition and distribution and may help to discover potential overseen diversity and new invasive species.

## Figures and Tables

**Table 1:** Sampling information.

Species	Strain	Location	Latitude and Longitude	LSU Accession No.	ITS Accession No.
*H. philippinensis* ribotype I	BGERL169	Tieshan Harbor	21°35′37″ N, 109°35′42″ E	OQ383330	OQ383353
*H. philippinensis* ribotype I	BGERL170	Lianzhou Bay	21°31′06″ N, 109°03′42″ E	OQ383331	OQ383354
*H. philippinensis* ribotype II	BGERL171	Lianzhou Bay	21°30′23″ N, 109°07′28″ E	OQ383332	OQ383355
*H. philippinensis* ribotype II	BGERL172	Lianzhou Bay	21°30′23″ N, 109°07′28″ E	OQ383333	OQ383356
*H. philippinensis* ribotype II	BGERL173	Lianzhou Bay	21°30′44″ N, 109°05′43″ E	OQ383334	OQ383357
*H. pseudotriquetra*	BGERL174	Tieshan Harbor	21°35′37″ N, 109°35′42″ E	OQ383335	OQ383358

## Data Availability

Data are available upon request, please contact the corresponding author Yixiao Xu.
